# Effect of Surface Treatment of Zirconia on the Shear Bond Strength of Resin Cement: A Systematic Review and Meta-Analysis

**DOI:** 10.7759/cureus.45045

**Published:** 2023-09-11

**Authors:** Roshni Kumar, Major Dhyanchand Singh, Vineet Sharma, Rahul Madaan, Kriti Sareen, Balwant Gurjar, Amit K Saini

**Affiliations:** 1 Prosthodontics, Rajasthan University of Health Sciences (RUHS) College of Dental Sciences, Jaipur, IND; 2 Dentistry, MDC Aurangabad, Aurangabad, IND; 3 Dentistry, Community Health Center, Raniwara, Jalore, IND; 4 Oral Medicine and Radiology, Rayat Bahra Dental College & Hospital, Mohali, IND

**Keywords:** meta-analysis, resin cements, shear bond strength, surface treatment, zirconia

## Abstract

This systematic review and meta-analysis aimed to evaluate the effect of different surface treatments on the shear bond strength (SBS) values between zirconia and resin cement compared to untreated specimens. The effects of various surface treatments on the bond strength between zirconia and resin cement were investigated by searching relevant articles on PubMed, ScienceDirect, and Google Scholar databases. A total of 13 studies that met the inclusion and exclusion criteria and addressed the research question were selected for statistical analysis. The studies were evaluated for heterogeneity, and a meta-analysis was performed.

In total, 13 in vitro studies were included in accordance with the eligibility criteria. All 13 studies consistently demonstrated that silica coating yielded the highest SBS, followed by sandblasting and laser treatments. The meta-analysis using a random-effect model indicated a significant intergroup comparison, except for a few studies.

Among the three treatments examined, the silica coating of zirconia was identified as the most effective in enhancing the bond strength between zirconia and resin cement. Further controlled laboratory and clinical studies are necessary to validate these findings and explore additional factors that may influence the effects of these surface treatments.

## Introduction and background

The successful outcome of polycrystalline ceramic restorations in clinical settings relies on the proper adhesion of dental elements. Adequate adhesion plays a crucial role in preventing microleakage, achieving better marginal adaptation, and increasing retention and fracture resistance [1]. Over time, fixed dental prosthesis materials have evolved from all-metal to porcelain-fused to metal restorations and now to all ceramics. The demand for improved physical characteristics in high-stress areas has led to the development of zirconia, a highly aesthetic all-ceramic restorative material with enhanced strength. This increase in the mechanical properties of zirconia is further accompanied by a reduction in the glassy matrix and silica content, resulting in acid-resistant ceramics [2,3].

Zirconia stands out from other dental ceramics due to its unique characteristic of transformation toughening [4]. When pure zirconia is heated to a temperature ranging from 1,470°C to 2,010°C and then cooled, it undergoes a phase transformation from a tetragonal phase to a monoclinic phase. This transformation is accompanied by a volume increase of approximately 3-5%, resulting in the development of high tensile stresses. These stresses can lead to the cracking of zirconia during the cooling process. Modification of zirconia by the addition of 3% of yttrium oxide is done by manufacturers to counter this problem.

The structural stabilization of zirconia by yttria results in a significant proportion of metastable tetragonal phase. This metastable tetragonal phase strengthens and toughens the structure by a localized transformation to the monoclinic phase when tensile stresses develop at crack tips. The resulting volume expansion adjacent to the crack tips produces a high local compressive stress around the crack tips, which increases the localized fracture toughness and inhibits the potential for crack propagation. The phenomenon of transformation toughening increases the flexural and tensile fracture resistance of stabilized zirconia prostheses and presumably the survival probabilities of zirconia-based restorations. The capability of stress-bearing of zirconia is directly proportional to transformation toughening. The mechanical properties, such as flexural strength and fracture resistance, are also greater than traditional ceramics.

However, a major concern associated with zirconia is the process of luting or bonding, as it lacks a silane phase and is not susceptible to etching with hydrofluoric acid, unlike glass ceramics [5,6]. In the case of single-unit zirconia restorations, the loss of retention is significantly higher compared to other etchable ceramic crowns [7].

Many authors have explored air-particle abrasion, also known as sandblasting, as a surface treatment method for zirconia. This technique involves roughening the zirconia surface, which increases surface area and enhances surface energy, resulting in improved micromechanical retention. However, it is important to note that the metastable nature of tetragonal zirconia can be affected by grit blasting, leading to a transition from the tetragonal to a monoclinic structure [8]. This structural change can decrease the strength of zirconia and increase its susceptibility to fracture. Specifically, the transformation capacity of zirconia during critical loading is reduced, which can have implications for the long-term durability of zirconia ceramics [5,6]. In addition to air-particle abrasion, another option for enhancing shear bond strength (SBS) involves laser treatment using various wavelengths, such as erbium-doped yttrium-aluminum-garnet (Er:YAG), neodymium-doped yttrium aluminum garnet (Nd:YAG), carbon dioxide (CO_2_), and erbium, chromium-doped yttrium, scandium, gallium and garnet (Er,Cr:YSGG) to condition ceramic surfaces before luting. Laser surface treatments have been found to increase the surface roughness, wettability, and SBS of the resin cement [1]. A study by Akyil et al. demonstrated that 2 W laser irradiation with Er,Cr:YSGG resulted in comparable surface roughness to airborne particle abrasion, while also achieving a stronger SBS compared to the control group [9].

Another method that has shown promising results is the tribochemical deposition of silica on the zirconia surface using air-blasting equipment. In this technique, the ceramic surface is initially abraded with aluminum oxide (Al_2_O_3_) particles to remove contaminants and create micro-roughness. Subsequently, the surface is subjected to airborne particle abrasion with aluminum trioxide particles modified by silica. The blasting pressure causes the silica-coated alumina particles to embed onto the ceramic surface, facilitating a chemical change induced by the silica [5,10]. Some studies have reported positive findings regarding SBS between resin and zirconia using this method [11,12]. However, it should be noted that other studies have observed a decrease in SBS after artificial aging [13-17]. This failure may be attributed to the lack of firm attachment between the silica coating and the zirconia surface [17]. SBS is used to measure the strength of the bond between two materials when a shear force is applied. The shear force is applied to the bonded area, which is the surface where the two materials are joined. The SBS is the maximum force that an adhesive joint can tolerate before fracture and is measured in megapascals (MPa).

Despite these various methods, the lack of a standardized treatment protocol makes the cementation of yttrium-stabilized tetragonal zirconia (Y-TZP) ceramic a challenge. This systematic review and meta-analysis was performed to evaluate different surface treatments of zirconia and their effect on the SBS values between the Y-TZP and resin cement compared to untreated specimens. The null hypothesis is that no significant difference will be found in the SBS values between the treated and untreated specimens.

## Review

Methodology

Sources

An electronic search was conducted to identify relevant articles published in the English language between 2000 and July 2020. The search was performed using databases such as PubMed, ScienceDirect, and Google Scholar. The search strategy employed a combination of Medical Subject Headings (MeSH) terms and keywords including “Zirconia surface treatment,” “All ceramic surface treatment,” “Bond strength of zirconia to resin cement,” “Bond strength of all ceramic to resin cement,” “Bond strength of zirconia to luting cement,” and “Bond strength of all ceramic to luting cement.”

Selection of Studies

The review process was conducted in two phases. In the first phase, the titles and abstracts obtained from the database search were screened by two authors to determine their relevance to the study. The full text of the abstracts that met the inclusion criteria was then obtained and evaluated. Any inconsistencies or disagreements were resolved through discussion between the authors or with the input of a third author.

Following the initial phase of the review process, a manual search was conducted on the selected articles as well as their references. In the second stage, the articles gathered were screened based on predefined inclusion and exclusion criteria. Relevant articles were identified and selected for further analysis and data extraction. Duplicates and articles lacking sufficient necessary data were excluded by the two authors, with any disagreements resolved through the suggestions of a third author.

During the article selection process, the inclusion criteria consisted of studies focusing on surface treatments such as laser, sandblasting with 50-micron alumina, and silica coating, along with the measurement of SBS. Additionally, studies with a sample size exceeding 150 were included. Conversely, review articles, studies published in Chinese, those with a sample size of less than 150, and articles assessing tensile strength were excluded from the analysis. The aim was to gather relevant studies that specifically examined the effects of these surface treatments on SBS.

Results

**Figure 1:**
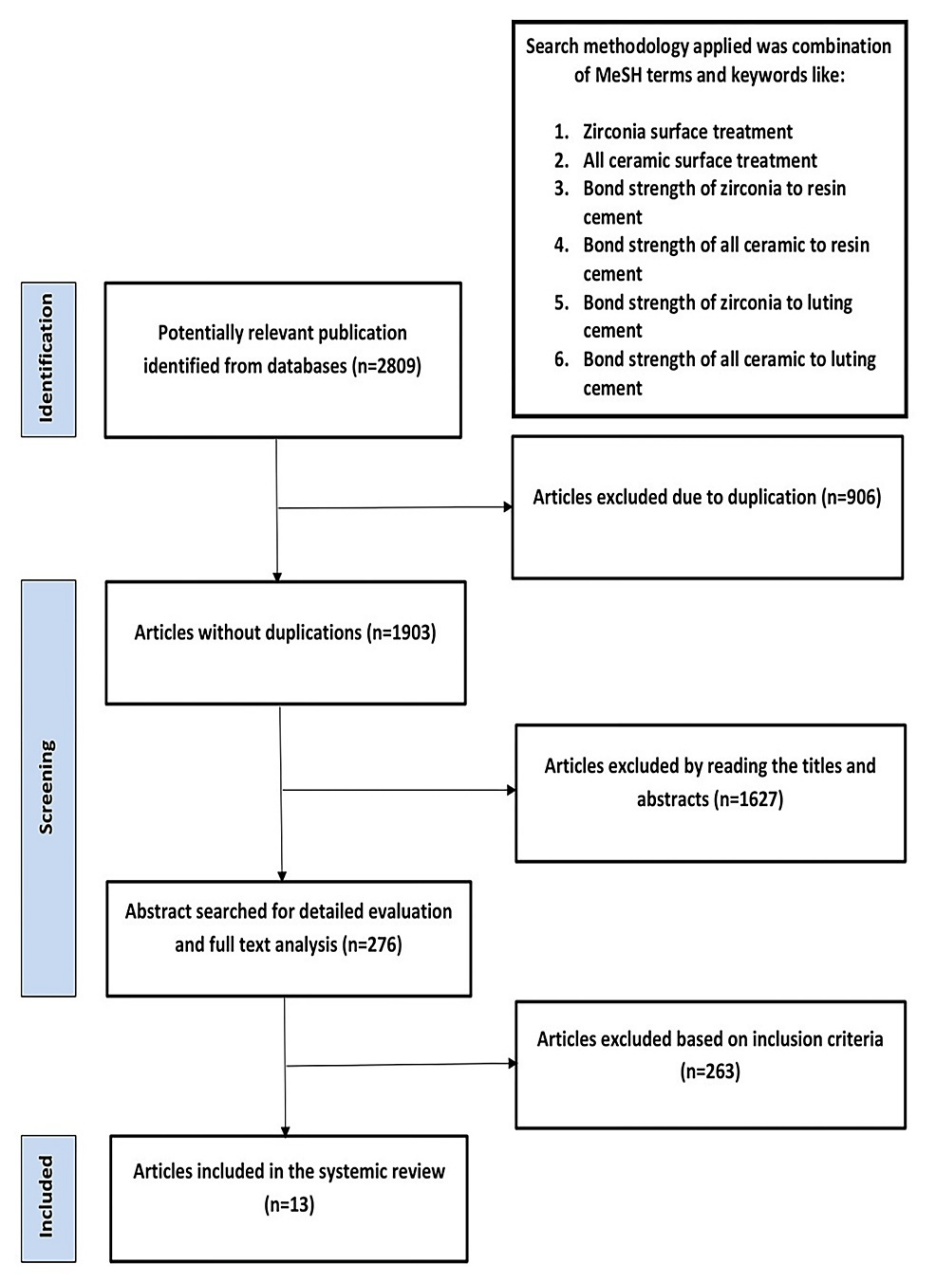
Preferred Reporting Items for Systematic Reviews and Meta-Analyses flowchart of the search strategy. n = number, MeSH = Medical Subject Headings

The initial electronic database search across PubMed/MEDLINE, ScienceDirect, and Google Scholar yielded a total of 2,809 titles. Following the screening of abstracts, 1,182 articles were identified as relevant and subsequently evaluated by two independent reviewers. Of the initial selection, 1,627 articles were deemed unrelated to the study and excluded. Through a thorough examination and discussions among the reviewers, 276 articles were chosen for full-text evaluation. After applying pre-screening, inclusion, and exclusion criteria and addressing the specific research question, a final set of 13 studies remained for further analysis (Figure 1).

Study Characteristics

Demographics and study outcomes: Initially, a total of 1,903 articles were identified based on the keywords related to zirconia surface treatment, all-ceramic surface treatment, sandblasting, laser, silica coating, and SBS. Among these, 1,627 articles were excluded as they did not meet the specified keyword criteria. Of the remaining 276 papers, further screening resulted in the selection of 13 clinical articles that encompassed a total of 2,878 specimens. Of these specimens, 1,366 underwent treatments involving sandblasting, silica coating, and lasers. Specifically, 618 (45.24%) specimens were treated with sandblasting, 330 (24.16%) with lasers, and 418 (30.61%) with silica coating. Each reviewer independently selected these specimens for inclusion in the final meta-analysis. We established a database into which we entered the information extracted from each article (Table 1). The outcomes of the meta-analysis with a fixed-effect model showed the level of heterogeneity among the studies (Table 2).

**Table 1:** Characteristics of the included study. n = number; MPa = megapascals; P < 0.05 = statistically significant; p > 0.05 = statistically non-significant

Authors	Country	Number of specimens	The technique of surface treatment used	Shear bond strength	Level of significance
Ozcan et al., 2003 [15]	Finland	216 (n = 36)	Air abrasion vs. silicon coating	Silica: 17.4 MPa (higher than air abrasion)	P < 0.001
Cavalcanti et al., 2009 [18]	Brazil	240 (n = 80 each)	Air abrasion vs. Er:YAG Laser	Air abrasion: 22.3–26.5 MPa Er:YAG Laser:15.8–23.0 MPa	P < 0.001
Nothdurft et al., 2009 [19]	Germany	360 (n = 30)	Air abrasion vs. silicon coating	Air abrasion: 4.29–23.88 MPa Silicon coating: 4.98–25.11 MPa	P < 0.001
Lin et al., 2010 [20]	Japan	210 (n = 10)	Air abrasion vs. silicon coating	Air abrasion: 15.32–50.91 MPa Silicon coating: 11.26–31.38 MPa	P < 0.05
Liu et al., 2013 [21]	Hong Kong	160 (n = 40)	Air abrasion vs. CO_2_ laser system	Air abrasion: 31.3 MPa CO_2_ laser system: -32.1 MPa	P > 0.05
Erdem et al., 2014 [22]	Turkey	200 (n = 50)	Air abrasion vs. silicon coating vs. Er:YAG laser system	Air abrasion: 9.9–14.8 MPa Silicon coating: 7.1–19.8 MPa Er:YAG laser: 0.0–5.0 MPa	P < 0.001
Liu et al., 2015 [23]	Hong Kong	30 (n = 6)	Air abrasion vs. silicon coating	Air abrasion: 25.17 MPa Silicon coating: 22.6 MPa	P > 0.05
Otani et al., 2015 [24]	Brazil	360 (n = 30)	Air abrasion vs. silicon coating	Air abrasion: 46.8 MPa Silicon coating: 9.2 MPa	P < 0.05
Aras et al., 2016 [1]	Brazil	160 (n = 20)	Air abrasion vs. silicon coating vs. Er:YAG laser system	Air abrasion: 2.5 MPa Silicon coating: 12.9 MPa Er:YAG laser: 1.2 MPa	P > 0.05
Iwasaki et al., 2016 [8]	Japan	352 (n = 176)	Air abrasion vs. silicon coating	Air abrasion: 2.3–8.1 MPa Silicon coating: 3–11.4 MPa	P < 0.05
Vicente et al., 2016 [25]	Spain	150 (n = 30)	Air abrasion vs. silicon coating vs. femtosecond laser	Air abrasion: 8.1 MPa Silicon coating: 9.5 MPa Femtosecond laser: 10.8 MPa	P < 0.05
Omidi et al., 2018 [26]	Iran	120 (n = 30)	Air abrasion vs. silicon coating vs. Er:YAG laser system	Air abrasion: 19.16 MPa Silicon coating: 22.21 MPa Er:YAG laser: 1.59 MPa	P < 0.05
Saade et al., 2020 [4]	Lebanon	320 (n = 80)	Air abrasion vs. Er,Cr:YSGG	Air abrasion: 28.3 MPa Er,Cr:YSGG laser: 25.9 MPa	P > 0.05

**Table 2:** Meta-analysis for shear bond strength. χ^2^ = chi-square coefficient; CI = confidence interval; df = degree of freedom; * = statistically significant; ** = statistically significant, I^2^ = percentage of heterogeneity (<50% in all parameters, showing lack of heterogeneity)

Shear bond strength	Heterogeneity test				Odds ratio	Relative risk	95% CI	Overall test	
	χ^2^	df	P-value	I^2^				Z-test	P-value
Air abrasion vs. silicon coating	21.01	6	1.019**	48%	5.31	4.46	7.90–11.774	0.128	0.021*
Air abrasion vs. laser system	16.24	3	0.391**	42%	1.65	0.51	1.34–9.521	3.791	0.062**
Air abrasion vs. silicon coating vs. laser system	8.91	4	0.078**	39%	3.90	1.92	3.25–21.21	2.915	0.004*

Characteristics of Included Studies

**Figure 2:**
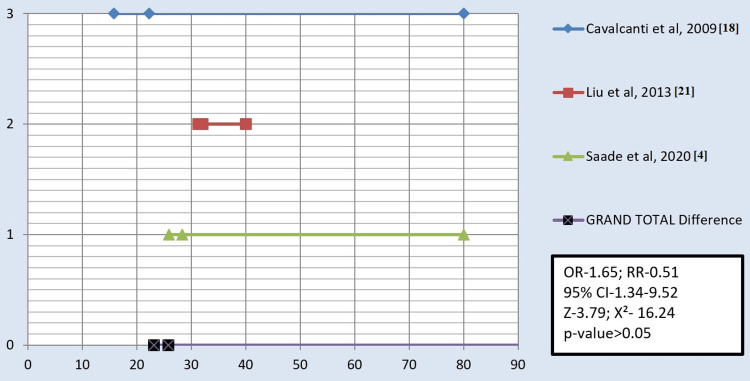
Forest plot depicting air abrasion versus laser. X-axis: Shear bond strength of air abrasion and laser along with the total samples (MPa). Y-axis: The sequence of studies in chronological order, with the grand total depicting the mean of all studies. OR = odds ratio; RR = relative risk; χ^2^ = chi-square coefficient; CI = confidence interval; Z = standard score

**Figure 3:**
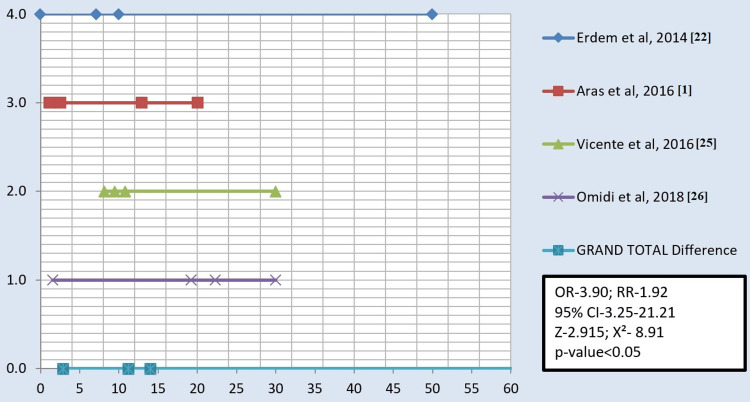
Forest plot depicting air abrasion versus silica coating versus laser. X-axis: Shear bond strength of air abrasion, silica coating, and laser along with total samples (MPa). Y-axis: The sequence of studies in chronological order, with the grand total depicting the mean of all studies. OR = odds ratio; RR = relative risk; χ^2^ = chi-square coefficient; CI = confidence interval; Z = standard score

**Figure 4:**
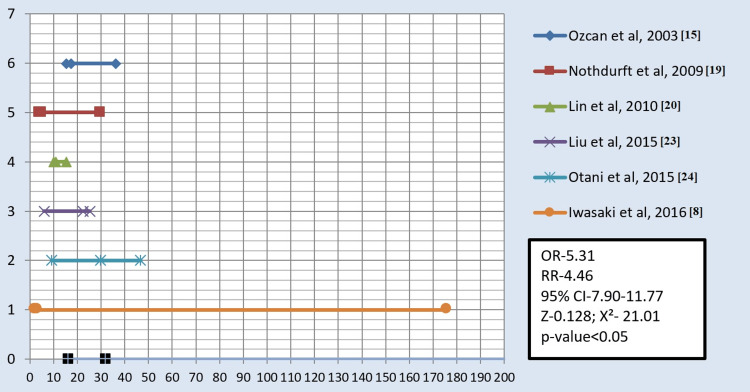
Forest plot depicting air abrasion versus silica coating. X-axis: Shear bond strength of air abrasion and silica coating along with total samples (MPa) Y-axis: The sequence of studies in chronological order, with the grand total depicting the mean of all studies. OR = odds ratio; RR = relative risk; χ^2^ = chi-square coefficient; CI = confidence interval; Z = standard score

The studies included in the analysis were conducted under in-vitro conditions and were prospective, cross-sectional studies. Various types of surface treatments on all-ceramic or zirconia materials were investigated. Among the included studies, three focused on comparing air abrasion with laser technique for the surface treatment of zirconia (Figure 2). Furthermore, four studies compared all three surface treatments, namely, sandblasting, laser, and silica coating (Figure 3). Additionally, six studies compared the effectiveness of air abrasion techniques with silica coating (Figure 4).

All authors in the selected studies compared the SBS after applying surface treatments using three different techniques, namely, sandblasting, silica coating, and laser treatment. The results showed that the SBS ranged from 2.5 MPa to 50.91 MPa for sandblasting, 4.98 MPa to 31.38 MPa for silica coating, and 0 MPa to 32.1 MPa for laser treatment. Across all 13 studies, it was consistently observed that the SBS was the highest with silica coating, followed by sandblasting and lasers. When comparing the three treatment groups, most studies found significant differences in SBS, except for a few studies conducted by Liu et al. in 2013 and 2015 [21,23], Aras et al. in 2016 [1], and Saade et al. in 2020 [4].

Discussion

Various techniques that mechanically promote resin-ceramic bonding have been documented to improve the SBS of luting cement to the ceramic surface. A well-established and recommended approach involves etching the inner surface of the restoration with hydrofluoric acid, followed by the application of a silane coupling agent [5]. This method has proven effective in roughening feldspathic ceramics, facilitating a stronger bond with resin. However, it is important to note that the use of hydrofluoric acid etching and silane treatment may not result in adequate bonding for certain new ceramics. This is particularly the case for high-strength ceramics and zirconia, as they lack the presence of the silicon dioxide (silica) phase necessary for hydrofluoric acid etching to be effective [25].

Saiji et al. found that the SBS between zirconia and resin cement was improved through airborne particle abrasion, regardless of the particle size or jet pressure used [27]. However, considering the potential impact of grit-blasting on the performance of zirconia restorations under continuous stresses in the oral environment, the use of laser as an alternative surface treatment method has gained interest and has been documented in numerous studies. Nd-YAG laser surface treatments have been shown to increase surface roughness, wettability, and SBS with resin cement [22].

The increasing demand for durable and aesthetically pleasing metal-free restorations has led to the widespread use of zirconia as a restorative material for single or multiple-unit fixed restorations. However, achieving a strong bond with luting materials can be challenging. To address this issue, alternative methods have been explored, including the deposition of silica on the bonding surface. One technique employed by various authors involves blasting alumina particles coated with silicon dioxide, followed by the application of silane binding agents (referred to as silicatization). The impact of these particles on the ceramic surface results in the deposition of a thin silica film. Silicatization not only creates micro-roughness on the surface but also chemically modifies it, thereby enhancing its bonding properties [19].

Different authors have explored the use of Er:YAG or CO_2_ lasers to enhance the adhesion between resin cement and zirconia [28]. This technique involves the removal of particles through micro-explosions and vaporization, known as ablation. However, the results regarding the effectiveness of lasers in improving the SBS between ZrO_2_ and resin cement are contradictory. The null hypothesis formulated in this study was rejected, as a significant difference in SBS values was observed between the luting cement and the treated and untreated surfaces. The results favored the surface-treated groups, indicating that the surface treatments had a positive impact on SBS.

The studies included in this meta-analysis were in vitro, prospective, cross-sectional studies. These studies investigated the effects of different surface treatments on all-ceramic or zirconia materials. Six studies compared the sandblasting or air abrasion technique with silica coating [7,15,19,20,23,24]. Three studies compared sandblasting/air abrasion with laser technique for the surface treatment of zirconia [4,18,21]. Additionally, four studies compared all three surface treatments (sandblasting, laser, and silica coating) together [1,25,26]. Across all included studies, it was consistently observed that the SBS was the highest with silica coating, followed by sandblasting and lasers.

## Conclusions

This systematic review and meta-analysis of in vitro studies suggests several key findings regarding the surface treatment of Zirconia. First, it was observed that such treatment significantly improves the SBS between zirconia and resin cement. Among the different treatment methods investigated, silica coating demonstrated the highest SBS, followed by sandblasting and lasers. The use of hydrofluoric acid etching and silane treatment is not effective for zirconia. Lasers as an alternative surface treatment method have gained interest due to their potential to reduce the negative impact of grit-blasting on the performance of zirconia restorations. However, further studies are needed to confirm these findings and investigate the long-term effects of different surface treatments on the performance of zirconia restorations.
